# Inter-Observer Agreement of a New Endoscopic Score for Ulcerative Colitis Activity: Preliminary Experience

**DOI:** 10.3390/diagnostics10040213

**Published:** 2020-04-12

**Authors:** Mariabeatrice Principi, Antonella Contaldo, Francesco Paolo Bianchi, Giuseppe Losurdo, Andrea Iannone, Enzo Ierardi, Silvio Tafuri, Alfredo Di Leo, IBD Endoscopy Team Work

**Affiliations:** 1 Section of Gastroenterology, Department of Emergency and Organ Transplantation, University “Aldo Moro” of Bari, 70124 Bari, Italy; contaldoantonella@gmail.com (A.C.); giuseppelos@alice.it (G.L.); ianan@hotmail.it (A.I.); ierardi.enzo@gmail.com (E.I.); alfredo.dileo@uniba.it (A.D.L.); 2 Department of Biomedical Science and Human Oncology, University of Bari “Aldo Moro”, 70124 Bari, Italy; frapabi@gmail.com (F.P.B.); silvio.tafuri@uniba.it (S.T.); 3 PhD Course in Organs and Tissues Transplantation and Cellular Therapies, Department of Emergency and Organ Transplantation, University “Aldo Moro” of Bari, 70124 Bari, Italy

**Keywords:** ulcerative colitis, disease activity, endoscopic scores, Extended Mayo Endoscopic Score

## Abstract

Ulcerative colitis (UC) endoscopic scores translate mucosal damage into values standardizing image analysis. Due to potential limits of current endoscopic activity indexes, we have elaborated on a new score, the “Extended Mayo Endoscopic Score (EMES),” and evaluated its inter-observer agreement in a multicenter endoscopy team, comparing concordance with the Mayo subscore. Sixteen UC consecutive patients underwent follow-up colonoscopy. Recorded videos were anonymously loaded on a web platform. Thirteen expert endoscopists evaluated UC activity using both Mayo and EMES. EMES was described in every colon segment: erythema (0: absent, 1: mild, 2: moderate, 3: severe), vascular pattern (0: normal, 1: reduction, 2: disappearance), erosions and ulcers (0: absent, 1: from 1 to 5, 2: 6 to 10, 3: >10). Weighted Fleiss’ kappa with 95% confidence interval (CI) and *p*-value defined inter-rater agreement. Global inter-observer agreement of EMES was moderate (kappa = 0.56, 95% CI = 0.46–0.67, *p* < 0.001). The evaluation of each colonic segment showed moderate agreement for all segments: ascending (kappa = 0.46, 95% CI = 0.32–0.60, *p* < 0.001), transverse (kappa = 0.48, 95% CI = 0.29–0.67, *p* < 0.001); descending (kappa = 0.49, 95% CI = 0.35–0.64, *p* < 0.001), sigmoid (kappa = 0.52, 95% CI = 0.39–0.65, *p* < 0.001) and rectum (kappa = 0.55, 95% CI = 0.42–0.69, *p* < 0.001). Mayo subscore agreement was similar to global EMES (kappa = 0.53, 95% CI = 0.39–0.66, *p* = 0.001). Therefore, our report emphasizes the importance of assessing inter-observer agreement for EMES, but also for other known scoring systems, including the Mayo subscore.

## 1. Introduction

The evaluation of disease activity in ulcerative colitis (UC) has undergone progressive development over the last 20 years through the characterization of clinical and laboratory parameters as well as endoscopic evaluation [1,2].

The advisable goal of defining inflammatory bowel disease (IBD) activity is to mark out a valid, objective and reproducible item representing a real mirror of the stage of illness [1,2]. In addition, a discrepancy between symptoms, laboratory data and severity of mucosal damage has often been the real limit to achieving this purpose [3]. The use of endoscopic scores is aimed at decoding the findings of mucosal damage and translating them into a numerical value, in order to allow standardization by reducing inter- and intra-observer variability [4].

In 1955, Truelove and Witts performed a first attempt to evaluate UC activity by hyperaemia and mucosal granularity [5] and, successively, Baron by bleeding severity [6]. Afterwards, further efforts regarding scoring included parameters reflecting the vascular picture, i.e., disappearance of submucosal vascular pattern, erythema and bleeding [7,8], as well as mucosal alterations, i.e., granularity, friability, erosions, muco-pus and ulcers [9,10]. More recently, Travis et al. introduced and validated a further score called the “Ulcerative Colitis Endoscopic Index of Severity” (UCEIS), which includes three variables: vascular pattern, bleeding, erosions/ulcers [11]. Nevertheless, the Mayo endoscopic subscore (Mayo), introduced by Schroeder in 1987, is the most widely used in clinical practice. Endoscopic rectal inflammation was graded on a 4-point scale (0–3) according to the following findings: (0) normal; (1) erythema, decreased vascular pattern, mild friability; (2) marked erythema, absent vascular pattern, friability, erosions; and (3) ulceration, spontaneous bleeding [12]. The Mayo subscore has the advantage of being simple and easy to use, although adequate training is required to reduce inter-observer variability [4]. Possible limitations of this score include the lack of defining the number and site of erosions and ulcerations. These parameters are known to be associated with disease outcome [13]. Furthermore, it does not provide data on disease activity in the different segments of the colon and does not consider the extent of the lesions, which is a very important recognized factor in establishing the disease course [14]. In this regard, a new complete assessment of mucosal damage has been proposed by means of the “Modified Mayo Endoscopic Subscore (MMES)”, in order to combine the simplicity of the Mayo subscore with its assessment along colonic segments showing active disease [15]. Despite its potential advantages, inter-observer concordance has not been evaluated and this point may constitute a limit to its diffusion and reliability. Other controversial methodological aspects of MMES may be the following: i. most of the patients were in clinical and endoscopic remission; ii. about a quarter of patients underwent a partial colonoscopy, although they were affected by a distal colitis; iii. an assessment of the number of and depth of ulcers was not provided [15].

The elaboration of this last scoring system, which considered some aspects of disease activity so far neglected, led us to develop a new revised endoscopic score, taking into account not only the extent of the disease, but also the number and site of erosive/ulcerative lesions. Therefore, the present study aimed to perform a preliminary basal evaluation of inter-observer agreement within a multicenter cross-sectional IBD Endoscopy Team Work use of the new endoscopic score for UC activity, i.e., the “Extended Mayo Endoscopic Score (EMES)” when compared with the conventional “Mayo subscore”.

## 2. Materials and Methods

### 2.1. Study Population and Data Collection

Ethical approval for this study was granted by the Local Ethics Committee (No. 0026148). Sixteen UC consecutive patients undergoing follow-up colonoscopy in a tertiary Gastroenterology Unit in January 2018 were enrolled for a cross-sectional study. Endoscopy was performed by a single operator (MP) and every patient signed their own informed consent. Endoscopic videos were recorded during the instrument withdrawal phase using a Sony HVO-500MD medical video recorder.

Anonymous videos were loaded on a multimedia platform which standardized communicative fluxes with a high level of rapidity and efficiency. In particular, the process was composed by:

- A model of the online page (SEM web service, FORMEDICA Scientific Learning srl, Lecce, Italy) allowing access to loaded case videos.
- The possibility to join with the multimedia platform through provision of a personalized username and password by IBD Endoscopy Team Work members.
- Access to a pre-filled page summarizing the characteristics and assessment methods for the Mayo subscore and EMES.

### 2.2. EMES

Unlike previous scores, EMES estimates disease activity in the different colonic segments (ascending, transverse, descending, sigmoid and rectum).

For each segment, according to their own opinion, every participant assigned in blind a numerical value related to the following features:

- erythema (0: absent, 1: mild, 2: moderate, 3: severe)
- submucosal vascular pattern (0: normal, 1: reduction, 2: disappearance)
- erosions (0: absent, 1: from 1 to 5 lesions, 2: from 6 to 10, 3: more than 10)
- ulcers (0: absent, 1: from 1 to 5 lesions, 2: from 6 to 10, 3: more than 10).

In detail, these parameters reflected the vascular picture (erythema and submucosal vascular pattern) and mucosal alterations (erosions and ulcers, i.e., loss of epithelial lining).

For each segment, a score from 0 to 11 was obtained, and the global score ranged from 0 to 55.

### 2.3. Agreement Assessment

IBD Endoscopy Team Work was composed of 13 participants with at least 5 years of endoscopic cross-sectional-specific experience from reference centers for IBD follow up and treatment in Southern Italy. Moreover, all were involved in regional and national IBD multicenter studies. The aim of the present study was to evaluate the basal inter-observer agreement, and no preliminary proper training was provided in the design of the study.

As above reported, all observers had the option to connect with a multimedia platform using a personalized username and password. For each video, a specific disease activity evaluation was obtained using both the Mayo endoscopic subscore and the new revised one (EMES); then these issues were recorded through access to a pre-filled page.

### 2.4. Statistical Analysis

In the present cross-sectional study, the inter-rater agreement was used in order to explain the inter-observer agreement of the Mayo score, global EMES, segment EMES and single item score of each segment, defining the weighted Fleiss’ kappa, with 95% confidence interval (CI) and the *p*-value. Significance was expressed as *p* < 0.05.

Kappa value ranged from −1 to 1, with zero value indicating statistical independence and 1 value indicating perfect agreement between observers. Different “agreement degrees” are expressed as follows: <0.00 poor, 0.00–0.20 slight, 0.21–0.40 fair, 0.41–0.60 moderate, 0.61–0.80 substantial, 0.81–1.00 almost perfect [16]. STATA MP 14 software was used for statistical analysis.

## 3. Results

### 3.1. Overall Characteristics of Patients

We enrolled sixteen consecutive UC patients, 5 males and 11 females, with a median age of 41.06 ± 17.18 years (range 18–69). The mean disease duration was 4.87 ± 3.26 years (range 1–10) with an average follow-up duration of 3.81 ± 2.27 years (range 1–7). Clinical activity of disease, evaluated using the full Mayo score, is demonstrated: remission phase in 3/16, mild in 4/16, moderate in 7/16 and severe activity in 2/16.

Endoscopic examination, uploaded on the web platform by the single operator performing the investigation (MP), is shown according to the extension of disease: six proctosigmoiditis, four left colitis and six pancolitis. The duration of the videos, recorded during the instrument withdrawal phase, was 10 ± 3 min (range 3–13).

### 3.2. EMES Agreement

The inter-observer agreement degree was moderate and statistically significant according to the global EMES (kappa = 0.56, 95% CI = 0.46–0.67, *p* < 0.001).

**Figure 1:**
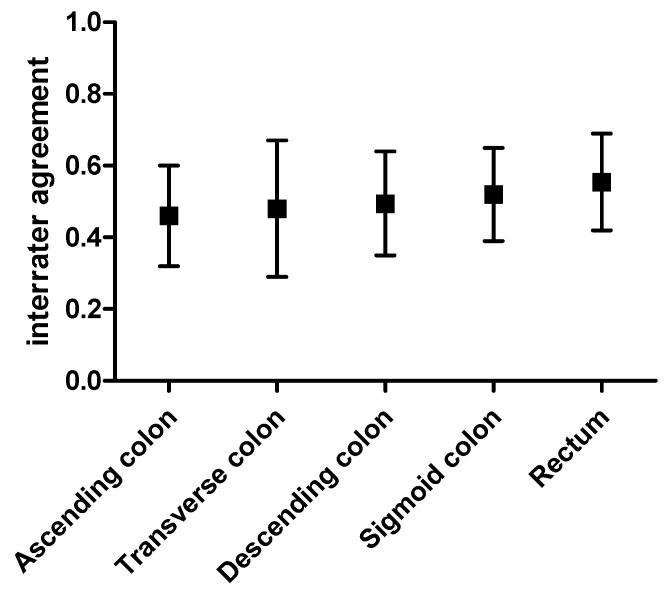
Inter-rater agreement on Extended Mayo Endoscopic Score (EMES) for each colon segment. Kappa values and 95% CI are represented.

Every colon segment showed the following results (Figure 1):

- ascending: kappa = 0.46, 95% CI = 0.32–0.60, *p* < 0.001
- transverse: kappa = 0.48, 95% CI = 0.29–0.67, *p* < 0.001
- descending: kappa = 0.49, 95% CI = 0.35–0.64, *p* < 0.001
- sigmoid: kappa = 0.52, 95% CI = 0.39–0.65, *p* < 0.001
- rectum: kappa = 0.55, 95% CI = 0.42–0.69, *p* < 0.001

Endoscopic evaluations according to EMES for each parameter of every colic segment showed the following results (Table 1):

- Ascending: erythema kappa = 0.39, 95% CI = 0.26–0.54, *p* < 0.001; erosions kappa = 0.31, 95% CI = 0.14–0.48, *p* < 0.001; submucosal vascular pattern kappa = 0.56, 95% CI = 0.39–0.74, *p* < 0.001; ulcers kappa = −0.01, 95% CI = −0.02–0.01, *p* = 0.335.
- Transverse: erythema kappa = 0.51, 95% CI = 0.30–0.72, *p* = 0.002; erosions kappa = 0.32, 95% CI = 0.10–0.54, *p* < 0.001; submucosal vascular pattern kappa = 0.48, 95% CI = 0.26–0.70, *p* < 0.001; ulcers kappa = 0.05, 95% CI = 0.02–0.08, *p* < 0.001.
- Descending: erythema kappa = 0.42, 95% CI = 0.26–0.59, *p* < 0.001; erosions kappa = 0.46, 95% CI = 0.30–0.61, *p* < 0.001; submucosal vascular pattern kappa = 0.49, 95% CI = 0.31–0.74, *p* < 0.001; ulcers kappa = 0.15, 95% CI = 0.00–0.30, *p* = 0.054.
- Sigmoid: erythema kappa = 0.44, 95% CI = 0.29–0.58, *p* < 0.001; erosions kappa = 0.51, 95% CI = 0.35–0.66, *p* < 0.001; submucosal vascular pattern kappa = 0.43, 95% CI = 0.28–0.60, *p* = 0.000; ulcers kappa = 0.36, 95% CI = 0.21–0.51, *p* < 0.001.
- Rectum: erythema kappa = 0.46, 95% CI = 0.30–0.61, *p* < 0.001; erosions kappa = 0.55, 95% CI = 0.37–0.74, *p* < 0.001; submucosal vascular pattern kappa = 0.55, 95% CI = 0.37–0.74, *p* < 0.001; ulcers kappa = 0.38, 95% CI = 0.24–0.53, *p* < 0.001.

**Figure 2:**
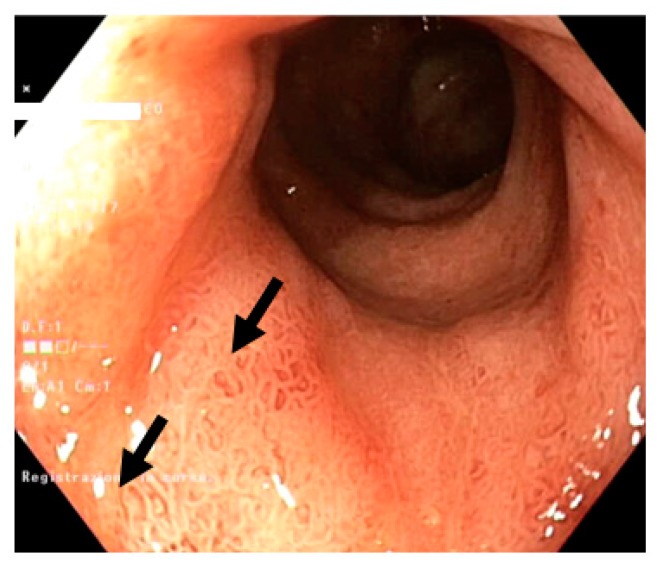
Erosions in the context of the hyperemic colonic mucosa (black arrows): superficial mucosal breaks within the hyperemic surrounding mucosa.

**Figure 3:**
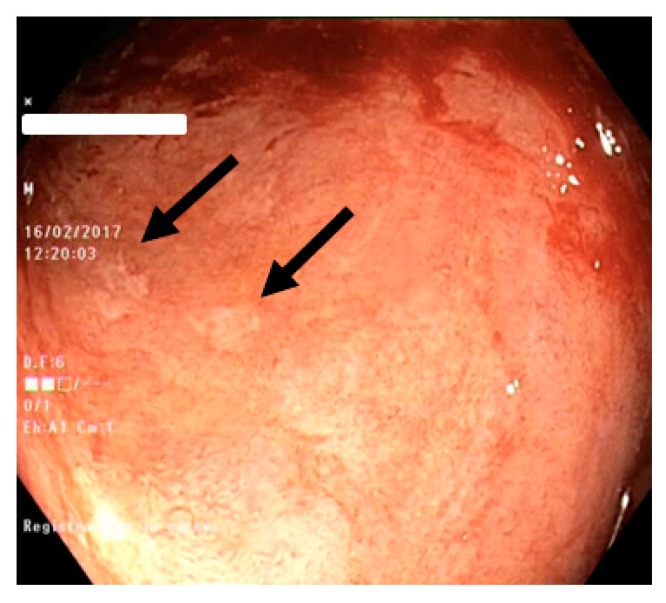
Ulcers in active ulcerative colitis (black arrows): mucosal breaks with fibrin spots within the hyperemic and friable mucosa and disappearance of the vascular pattern.

Of relevance, the only lack of significance was seen for erosions (Figure 2) and ulcers (Figure 3) in the ascending and only for ulcers in the descending colon. In detail, ulcers and erosions were found only in patients with moderate and severe disease activity, i.e., 9 out of 16 patients (56.2%).

### 3.3. EMES Versus Mayo Score

**Figure 4:**
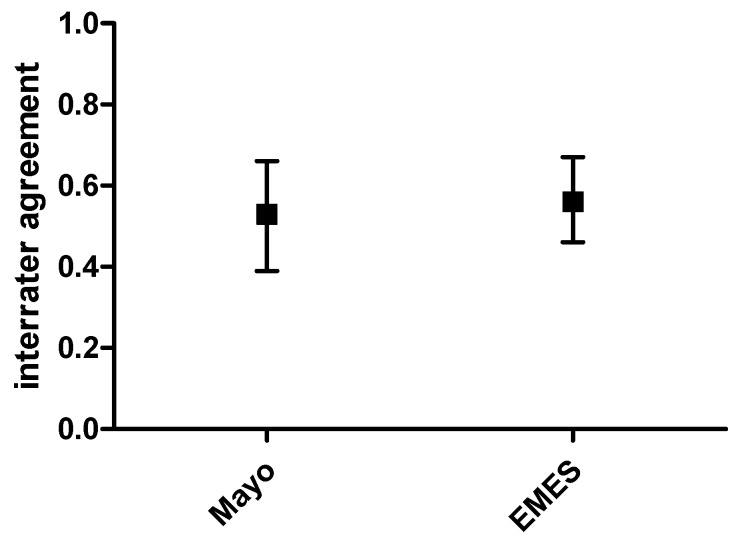
Inter-rater agreement on EMES and Mayo scores. Kappa values and 95% CI are represented.

Inter-observer agreement was moderate and statistically significant even when evaluated according to the Mayo subscore (kappa = 0.53, 95% CI = 0.39–0.66, *p* < 0.001). The result was similar to that reported above for EMES global agreement (Figure 4).

The values demonstrated a concordance in the assessment of disease activity between the two scoring systems in the case of pancolitis in the severe activity phase (Supplementary Material Video S1). In the illustrated case, the average value of the Mayo endoscopic subscore was 2.84 ± 0.37 and the EMES was 28.30 ± 5.40.

Discrepancy between the two scoring systems reflected a limited extent of the disease (Supplementary Material Video S2) in the case of proctosigmoiditis in the moderate activity phase. In the illustrated case, the average value of the Mayo endoscopic subscore was 1.61 ± 0.50 and EMES score was 6.46 ± 3.59.

## 4. Discussion

The first step in the present study was the development of a new revised endoscopic score for UC activity, i.e., the “Extended Mayo Endoscopic Score (EMES)”. The prerequisite of this revised system of disease activity scoring was stimulated by two main possible limitations of the Mayo subscore, the most used score in clinical practice, i.e., the lack of defining the number and depth of ulcerations. These last features may be of relevant interest, since they are associated with disease outcome [14,15]. A further potential advantage of the new revised score is the possibility of providing data on disease activity in the different segments of the colon, thus allowing the evaluation of another significant issue to establish disease course, i.e., the extent of the lesions [15,17,18]. This parameter may even reflect the possible progression of the disease towards the proximal direction, which is a reliable sign of its worsening [17,18]. Moreover, endoscopic scores, other than predicting clinical course and short- and long-term outcomes, could, at times, guide therapeutic decisions [19]. In this regard, a recent study by de Jong et al. [20] highlighted this aspect by demonstrating that an UCEIS score ≥4 was significantly associated with treatment escalation, and this cutoff could, therefore, be used to support clinical decisions. On the other hand, endoscopic intestinal damage regression with the achievement of so-called “mucosal healing” (MH) has become an important “treat-to-target” parameter in UC, since it reduces the risk of exacerbations, hospitalization, colorectal cancer and colectomy [21,22,23,24]. However, there is no unanimous definition of MH. In most clinical trials, a suitable goal of therapy is represented by only the improvement of the endoscopic lesions with the feature of “partial MH” [25], Mayo subscore 1, despite the fact that some studies have used a more strict definition of complete mucosal healing, Mayo 0 [26].

The second step of the present study was to evaluate the feasibility of the revised score by preliminary experience within a multicenter team, composed of 13 participants with at least 5 years of endoscopic experience from reference centers for IBD treatment in Southern Italy. Therefore, the aim of the study was to evaluate the inter-observer agreement and compare it with that of the endoscopic Mayo subscore.

A final interesting aspect of this preliminary experience was represented by the possibility of all participants to access to a multimedia platform, which had been specifically realized with the aim of assisting not only the video viewing of each endoscopic examination, but also the assignment of both the Mayo subscore and the new revised endoscopic score, i.e., the object of the present study. This computed procedure speeds up the appliance of endoscopic scores during the investigation report, thus avoiding the need to spend an excessive time that could hamper the process in clinical practice. Endoscopic videos were recorded during the instrument withdrawal phase. The duration of the videos was 10 ± 3 min (range 3–13). European society of gastrointestinal endoscopy (ESGE) guidelines [27] indicate a withdrawal time of at least 6 min in 90% of screening colonoscopies as a quality standard; of course, the period was longer in this study for a detailed evaluation of activity disorder.

Feagan et al. emphasized the essential role of central review of the endoscopic images in multicentric studies [28]. In this regard, a singular experience was performed by Stidham et al. [29] through the use of 16,514 images from 3082 patients with UC undergoing colonoscopy. Using this modality, a 159-layer convolutional neural network was realized as a learning model. In comparison, two independent observers were supported by a third reviewer in the case of score discrepancies. This study demonstrated that the learning model performance was similar to experienced human reviewers in grading UC endoscopic severity by Mayo subscore. Since this software system could be modified in the case of changes in the size or type of processed data, the authors concluded that it could improve the use of colonoscopy in both research and routine practice.

Mayo subscore reproducibility has been demonstrated in two foremost studies [30]. Osada et al. highlighted substantial agreement (k value 0.74) among four expert endoscopists, even if it was moderate (k value 0.46) among four trainee operators [31]. Daperno et al. confirmed this finding, showing for Mayo subscore a moderate agreement (k value 0.53, 95% CI = 0.47–0.56) among 14 gastroenterologists with expertise in clinical and endoscopic management of IBD [32]. A similar result was observed in the present study, where the weighted Fleiss’ kappa value indicated moderate agreement for global and segmental EMES, which appeared to be similar to the value found for the Mayo subscore. The different agreement results between the study of Osada and that of Daperno, as well as the similarity of our findings with those of the latter report, could be explained by the number of participants. It is likely that a large number of operators, even if necessary for an accurate agreement evaluation, might have induced a scattering of the single values of assigned scores.

Nevertheless, the *p*-value was significant in our study, thus indicating the potential feasibility of the revised scores in the general population of our geographical area. Despite the encouraging overall results of this study, a clear disagreement was observed for ulcer evaluation in the ascending and descending colon. A possible explanation for this result may be due to the lack of the habit to verify the number of these lesions, despite their potentially easy recognition by expert operators. It is, moreover, possible that the low agreement for ulcer identification in the ascending and descending colon could be attributed to the difficulty in differentiating these two entities in some borderline situations. This issue has been pointed out by de Lange et al. [33]. In addition, in our series, ulcers and erosions were found only in patients with moderate and severe disease activity, i.e., 9/16 patients (56.2%), and the low frequency could have affected the inter-observer agreement. A conclusive remark about this issue might be that a limitation of the Mayo subscore is that it does not recognize as different a patient with only a small ulcer and another one with multiple large ulcerations, and EMES has been planned to overcome this burden. Paradoxically, the limitation of EMES appeared to be that the agreement for ulcers could be unsatisfactory in some conditions. However, it differed from the Mayo subscore in that it was still efficient to differentiate the severity of overall intestinal damage, as illustrated in the comparison of the two videos (Supplementary Materials). Finally, regardless of an overall slight margin of agreement, a kappa coefficient <0.40 was seen for all parameters except for submucosal vascular pattern in the ascending colon. This isolated finding may be presumably due to the poor attitude toward intra-observer comparison even by expert operators. Indeed, endoscopists participating in this study, although with experience in the field of IBD, are not used to comparing their investigation reports with those of other operators, since their activity involves carrying out their work individually and not in a shared modality.

## 5. Conclusions

In conclusion, this preliminary study suggests that the use of a new revised endoscopic score, which is skilled to define the activity of the UC, taking into account relevant issues poorly considered until now, may be feasible. However, the data obtained highlights the need to further improve the margin of agreement. The number of different proposed scores emphasizes that objective endoscopic scoring systems for UC are difficult to attain, especially with acceptable inter-observer agreement. In a way, our attempt to find a modified grading system to help with this problem showed that inter-observer agreement was almost satisfactory for the score except with elementary lesions, especially those regarding loss of epithelial substance, i.e., erosions and ulcers. Nevertheless, it represents an encouraging starting point to continue the search for a better tool for UC endoscopic grading, since it highlights the need for dedicated training to achieve this goal. In addition, the use of pertinent multimedia platforms could be the most appropriate instrument for accustoming operators to the use of endoscopic scores and comparing their evaluations with that of other professionals in order to standardize imaging interpretation and optimize agreement. In this regard, this instrument might be used for both preliminary training and successive agreement studies.

## Figures and Tables

**Table 1:** Inter-observer agreement on EMES for each parameter of each colonic segment.

Parameters	Kappa	95% CI	*p*
**Ascending**			
**Erythema**	0.39	0.26–0.54	<0.001
**Erosions**	0.31	0.14–0.48	0.001
**Submucosal vascular pattern**	0.56	0.39–0.74	<0.001
**Ulcers**	−0.01	−0.02–0.01	0.335
**Transverse**			
**Erythema**	0.51	0.30–0.72	<0.001
**Erosions**	0.32	0.10–0.54	<0.001
**Submucosal vascular pattern**	0.48	0.26–0.70	<0.001
**Ulcers**	0.05	0.02–0.08	<0.001
**Descending**			
**Erythema**	0.42	0.26–0.59	<0.001
**Erosions**	0.46	0.30–0.61	<0.001
**Submucosal vascular pattern**	0.49	0.31–0.74	<0.001
**Ulcers**	0.15	0.00–0.30	0.054
**Sigmoid**			
**Erythema**	0.44	0.29–0.58	<0.001
**Erosions**	0.51	0.35–0.66	<0.001
**Vascular pattern**	0.43	0.28–0.60	<0.001
**Ulcers**	0.36	0.21–0.51	<0.001
**Rectum**			
**Erythema**	0.46	0.30–0.61	<0.001
**Erosions**	0.55	0.37–0.74	<0.001
**Submucosal vascular pattern**	0.55	0.37–0.74	<0.001
**Ulcers**	0.38	0.24–0.53	<0.001

## Supplementary Materials

The following are available online at https://www.mdpi.com/2075-4418/10/4/213/s1.

## Appendix A

**IBD Endoscopy Team Work:** Francesca Albano (Division of Gastroenterology, Veris Delli Ponti Hospital, Scorrano, Italy), Leonardo Allegretta (Division of Gastroenterology, Santa Caterina Novella Hospital, Galatina, Italy), Fabrizio Bossa (Division of Gastroenterology, Casa Sollievo della Sofferenza, IRCCS, San Giovanni Rotondo, Italy), Nicola De Tullio (Division of Gastroenterology, Hospital F. Miulli, Acquaviva delle Fonti, Italy), Nicola Della Valle (Gastroenterology Unit, Department of Medical Sciences, University of Foggia, Foggia, Italy), Sara Fiorella (Division of Gastroenterology and Digestive Endoscopy, Padre Pio Hospital, Vasto, Italy), Yusef Hadad (Division of Internal Medicine, Card. Panico Hospital, Tricase, Italy), Silvia Mazzuoli (Gastroenterology Unit, San Nicola Pellegrino Hospital, Trani, Italy), Primaldo Paiano (Division of Gastroenterology, Veris Delli Ponti Hospital, Scorrano, Italy), Antonio Rispo (Gastroenterology, Department of Clinical Medicine and Surgery, School of Medicine “Federico II” of Naples, Naples, Italy), Elisa Stasi (Gastroenterology Unit, “Vito Fazzi” Hospital, Lecce, Italy), Antonio Tursi (Gastroenterology Service, ASL BAT, Andria, Barletta-Andria-Trani, Italy).
